# Depletion of Microglia Increases Cortical Oligodendrocyte Density During Remyelination

**DOI:** 10.1002/glia.70120

**Published:** 2026-01-08

**Authors:** Hannah Katherine Loo, Joseph Gallegos, Christine Mialki, Gregory E. Perrin, Thomas Malloy, Jennifer L. Orthmann‐Murphy

**Affiliations:** ^1^ Neuroscience Graduate Group, Perelman School of Medicine University of Pennsylvania Philadelphia Pennsylvania USA; ^2^ Department of Neurology, Perelman School of Medicine University of Pennsylvania Philadelphia Pennsylvania USA

**Keywords:** cortex, cuprizone, microglia, oligodendrocytes, remyelination

## Abstract

Cortical demyelination is a critical contributor to progressive disease in multiple sclerosis (MS). The barriers to cortical remyelination following demyelination are not fully understood, and there are no remyelinating treatments for MS. We previously took advantage of the spatial and temporal resolution of longitudinal in vivo imaging to study cortical oligodendrocyte regeneration following cuprizone‐induced demyelination and found that oligodendrocyte regeneration was impaired. In this study, we investigated whether cortical reactive microglia disrupt oligodendrocyte regeneration. To do so, we used a combination of in situ RNA and immunofluorescence labeling to characterize cortical microglia reactive states following cuprizone‐mediated demyelination. We then depleted cortical microglia by administering a Csf1r inhibitor during the recovery period from cuprizone and quantified oligodendrocyte recovery. We found that following cortical demyelination, deep cortical microglia change morphology, downregulate homeostatic markers (P2RY12, TMEM119), and upregulate a marker (CD68) associated with activated macrophages. These reactive changes persisted through early recovery post‐cuprizone but resolved by late recovery. Depleting cortical microglia post‐cuprizone restored the baseline density of deep cortical ASPA+ oligodendrocytes at early and late recovery. There were also more deep cortical BCAS1+ differentiating oligodendrocytes at early recovery when microglia were depleted, suggesting that transient deep cortical reactive microglia impair oligodendrocyte differentiation following demyelinating injury. Together, we found that cortical microglia adopt spatially restricted reactive functions after demyelination and deep cortical reactive microglia transiently reduce differentiating oligodendrocytes. A potential therapeutic strategy for progressive MS could involve targeting transiently reactive microglia at the right time and place in cortical lesions to promote oligodendrocyte regeneration.

## Graphical abstract

- Cuprizone demyelination induces transient reactive states in deep cortical microglia (MG) through early recovery.
- Depleting MG in recovery increases the number of deep cortical mature/pre‐myelinating oligodendrocytes.
- Deep cortical reactive MG reduce oligodendrocyte density after demyelination.

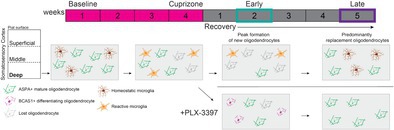

## 1 Introduction

Multiple sclerosis (MS) is an inflammatory demyelinating disorder characterized by white and gray matter lesions in the central nervous system (CNS; Reich et al. 2018). Following oligodendrocyte and myelin loss, remyelination occurs when oligodendrocyte precursor cells (OPCs) differentiate into new oligodendrocytes (Neely et al. 2022). Our understanding of the barriers to remyelination, particularly in cortical demyelinating MS lesions, is limited. Pathology studies of gray matter regions show that demyelination can occur early in MS, and cortical lesion burden correlates with signs of progressive disease, such as cognitive deficits and brain atrophy on imaging (Zivadinov et al. 2024). However, because pathology represents a single point in time, and gray matter lesions are not detectable on standard clinical MRI, we do not yet understand the temporal relationship between cortical lesions and the onset and progression of symptoms. These challenges underscore the need for experimental systems to study the dynamic mechanisms underlying cortical demyelination and recovery. To overcome these limitations, we previously combined the cuprizone model of demyelination with longitudinal in vivo two‐photon imaging in a transgenic mouse line to track the fate of mature cortical oligodendrocytes through recovery (Orthmann‐Murphy et al. 2020). We found that oligodendrocyte regeneration in the somatosensory cortex is incomplete, and this is driven primarily by inefficient oligodendrocyte replacement in deep cortical regions. For the current study, we adapted our previous approach to determine whether cortical microglia are a barrier to oligodendrocyte recovery in the deep cortex.

Microglia are the resident innate immune cells of the brain (Ginhoux et al. 2013). Over the course of development, microglia have many critical homeostatic functions (Li and Barres 2018), including directly influencing the formation of oligodendrocytes and myelin in white matter regions during early postnatal development (Irfan et al. 2022). Microglia are also important for the maintenance of healthy myelin in the adult brain (McNamara et al. 2023). In the setting of demyelinating injury, microglia remove myelin debris by phagocytosis (Yamasaki et al. 2014), thereby eliminating at least one barrier to OPC differentiation (Kotter 2006; Plemel et al. 2013). However, the role of microglia in oligodendrocyte degeneration and regeneration is more complex. Depletion of microglia, using an inhibitor of colony stimulating factor 1 receptor (Csf1r) either before or during cuprizone administration, led to delayed or reduced demyelination in the corpus callosum (Marzan et al. 2021). This suggests that reactive microglia play an important role in cuprizone‐induced demyelination and oligodendrocyte loss, but it is unknown how they contribute to the formation of replacement oligodendrocytes in gray matter. In the lysolecithin model of white matter demyelination, microglia can secrete pro‐regenerative factors during recovery time points that enable OPC differentiation and oligodendrocyte regeneration (Miron et al. 2013). However, microglia are uniquely attuned to their local environment, and within the healthy cortex exhibit spatially heterogeneous gene expression (Hammond et al. 2019; Paolicelli et al. 2022; Tarozzo et al. 2003). We hypothesized that cortical microglia adopt spatially heterogeneous reactive responses following demyelinating injury that could differentially influence oligodendrocyte regeneration.

Here, we show that superficial and deep cortical microglia differentially respond to cortical demyelination. Following cuprizone administration, deep cortical microglia changed morphology, downregulated homeostatic markers, and upregulated CD68, forming a reactive state that persists through early recovery. Depleting cortical microglia during post‐cuprizone recovery, with the administration of a Csf1r inhibitor, unexpectedly restored mature ASPA+ oligodendrocyte density to baseline levels in the deep cortex during early recovery and through late recovery. In addition, during early recovery, there were also more pre‐myelinating BCAS1+ differentiating oligodendrocytes in the deep cortex after microglia were depleted. These results suggest that deep cortical *reactive* microglia are a barrier to oligodendrocyte regeneration following demyelination.

## 2 Methods

### 2.1 Animal Care and Use

Adult mice of both sexes were used for experiments and randomly assigned to experimental groups. All mice were healthy and did not display any overt behavioral phenotypes, and no animals were excluded from the analysis. C57BL/6 mice were obtained from Charles River. Generation and genotyping on Mobp‐eGFP (Gensat; Hughes et al. 2018) and Cx3Cr1‐eGFP mice (Jax #00582; Jung et al. 2000) were previously described. In brief, mouse ear tissue was collected, digested, and amplified by PCR with strain‐specific primers before being run on a 2% agarose gel, and resulting bands were imaged to determine genotype. Mice were maintained on a 12 h light/dark cycle, housed in groups no larger than 5, and food and water were provided *ad libitum* (except during cuprizone administration, see below). All animal experiments were performed in strict accordance with protocols approved by the Institution of Animal Care and Use Committee at the University of Pennsylvania (Philadelphia, PA).

### 2.2 Cuprizone Administration

At 8–12 weeks of age, male and female mice were fed a diet of milled, irradiated 18% protein rodent diet (Teklad Global) alone (sham) or supplemented with 0.2% w/v bis(cyclohexanone) oxaldihydrazone (Cuprizone, Santa Cruz Biotechnology) for 4 weeks, and then returned to a standard pellet diet as previously described (Baxi et al. 2017; Orthmann‐Murphy et al. 2020).

### 2.3 Csf1r Antagonist Administration

After mice were fed a sham or cuprizone‐supplemented diet for 4 weeks, mice were fed a diet of either PLX‐3397 (290 ppm; Pexidartinib, SelleckChem and Research Diets) or PLX‐5562 (1200 ppm; SelleckChem and Research Diets) pellets for 2 or 5 weeks.

### 2.4 Immunohistochemistry

Mice were deeply anesthetized with sodium pentobarbital (100 mg/kg body weight) and perfused transcardially with 4% paraformaldehyde (PFA; in 0.1 M phosphate buffer, pH 7.4). Brains were extracted and then post‐fixed in 4% PFA for 18–24 h before being transferred to a 30% sucrose solution (in PBS, pH 7.4). Tissue was stored at 4°C for more than 48 h before preparing for cryosectioning. Brains were then embedded in OCT, and sectioned at 34 μm thickness on a cryostat (Leica CM 1950 Cryostat) at −20°C. Immunofluorescence staining was performed on free‐floating sections. Sections were pre‐incubated in blocking solution (5% normal donkey serum, 0.3% Triton X‐100 in PBS, pH 7.4) for 1 h at room temperature, then incubated for 24 h at 4°C in primary antibody (listed in Appendix S1, Key Resources). Secondary antibody (see Appendix S1) incubation was performed at room temperature for 2 h. Sections were mounted on slides with Aqua Polymount (Polysciences). Images were acquired using an epifluorescence microscope (Zeiss AxioImager M2) with Zen 3.0 software.

### 2.5 In Situ RNA Labeling

Mice were perfused with PBS as described above. Brains were extracted and embedded in OCT, sectioned at 15–20 μm thickness on a cryostat (Leica CM 950 Cryostat) at −20°C and mounted onto slides. Sections were then fixed, dehydrated, and sealed with a hydrophobic barrier. Preparation, hybridization of probes, and development of fluorescent signal were performed in accordance with the ACD RNAscope Multiplex Fluorescent Reagent Kit v2 Assay protocol (Document Number 323100‐USM).

### 2.6 Image Processing and Analysis

Immunofluorescence image Z‐stacks were acquired using a 10× objective, with a slice interval of 0.5 μm (for cell counts) or 3 μm (for myelin quantification), and compressed into maximal intensity projections representing a maximal thickness of up to 6.5 μm for cell counts or 15 μm for myelin quantification, respectively (ImageJ). For in situ RNA labeled sections, images were acquired as Z‐stacks using a 20× objective (Zeiss). Image stacks were tiled and stitched together using DAPI as a reference channel (Zeiss Zen software). Stitched images were then processed as .tif files and compressed into maximal intensity projections of up to 4.5 μm (ImageJ).

Coronal images were then rotated and cropped to yield a 500 μm × 750 μm region with the pial surface of somatosensory cortex aligned to the top. For cell counts, a blinded observer quantified the number of NG2+, GFAP+, Iba1+, ASPA+, BCAS1+, and GFP+ cells within each 500 μm × 750 μm region using a custom counting macro in FIJI/ImageJ (Orthmann‐Murphy et al. 2020). These regions were then subdivided into 500 μm × 250 μm zones relative to the pial surface, designating superficial (0–250 μm), middle (250–500μm), and deep (500–750 μm) cortical regions. Microglia were identified with either Iba1 (in Mopb‐eGFP mice or C57BL/6 WT littermate controls) or GFP (in Cx3cr1‐eGFP mice), and we then determined whether these cells were also positive for a microglia state marker (P2RY12 or CD68). For RNA expression analysis, a blinded observer quantified the number of cells containing TMEM119+ RNA puncta within a 500 μm × 750 μm region, and then these regions were subdivided into 500 μm × 250 μm zones relative to the pial surface as above. For myelin quantification, the percentage of MBP signal within 500 μm ×750 μm somatosensory cortical regions was obtained using a custom macro that divides the image into superficial, middle, and deep regions, and then uses the ‘auto‐threshold’ tool with Otsu parameters to define MBP+ signal (ImageJ). For representative images, brightness and contrast levels were adjusted for clarity.

### 2.7 Morphology Analysis

To quantify changes in microglial morphology, a blinded observer categorized Iba1+ (in Mobp‐eGFP or WT littermates) or GFP+ microglia (in Cx3Cr1‐egfp mice) as either homeostatic (small, circular cell body with ramified processes) or reactive (larger and amoeboid cell body with short and thick processes). To quantify BCAS1+ cell morphology, a blinded observer categorized BCAS1+ cells as either ‘early‐myelinating’ (possessing multiple processes) or ‘pre‐myelinating’ (possessing few processes) as previously described (Fard et al. 2017; Bergner et al. 2024). All counts were performed within a 500 μm × 750 μm region using a custom counting macro (ImageJ).

### 2.8 Statistical Analysis

Statistical analyses were performed with Prism (GraphPad). Significance was determined using a one‐way ANOVA test with Tukey correction for multiple comparisons, unless otherwise specified. *N* represents the number of animals used in each experiment. Data are reported as mean ± SEM and *p < 0.05; ** = *p* < 0.01; *** = *p* < 0.001; **** = *p* < 0.0001.

All key reagents are summarized in Appendix S1.

The graphical abstract was generated in part using Biorender images: Created in BioRender. Orthmann Murphy, J. (2026) https://BioRender.com/ln2ul7a.

## 3 Results

### 3.1 Cortical Oligodendrogenesis Is Impaired Following Cuprizone‐Induced Demyelination

**FIGURE 1:**
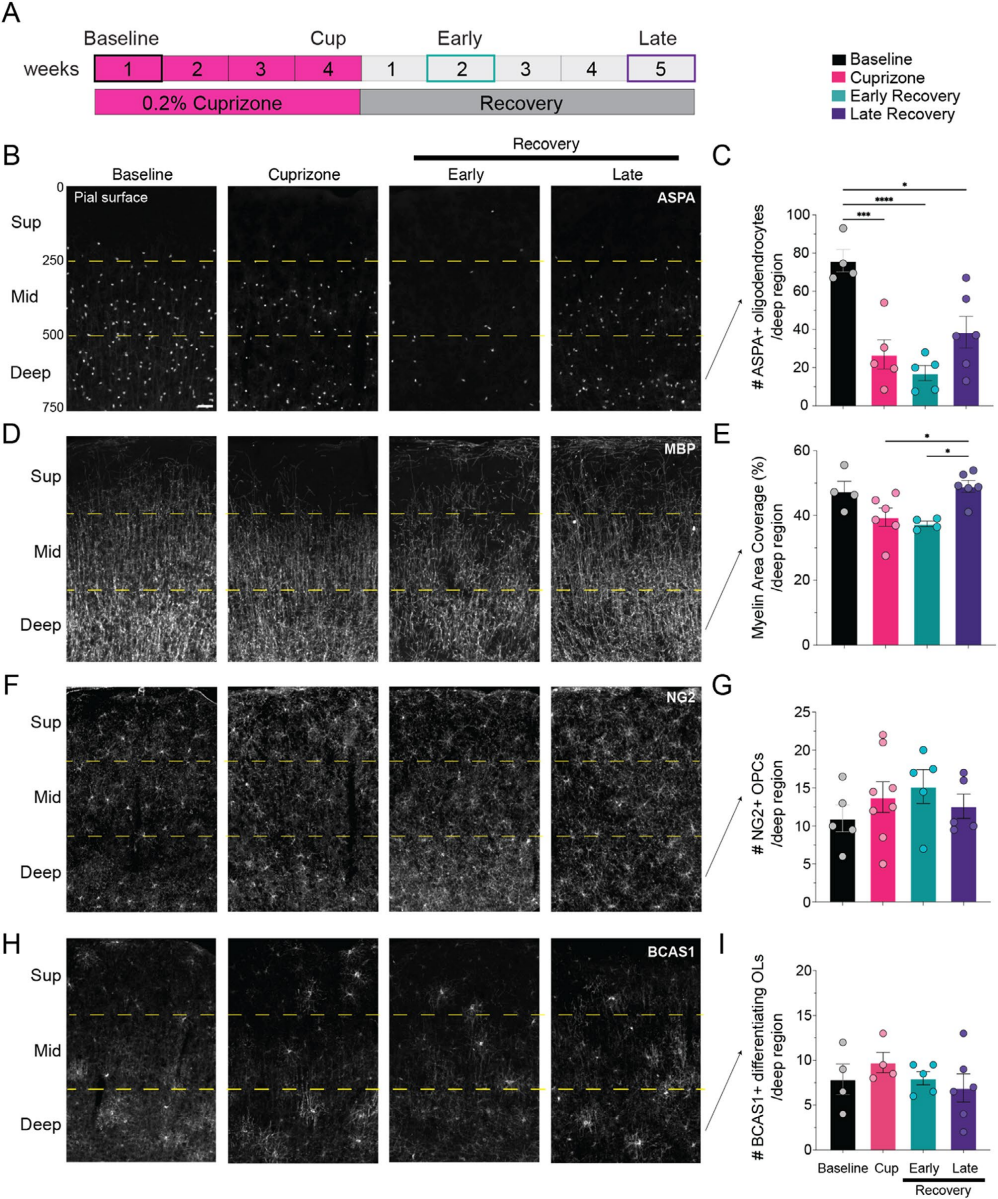
Replacement of deep cortical oligodendrocytes is impaired after demyelination. (A) Experimental paradigm. (B, D, F, H) Representative coronal images of cortical ASPA+ oligodendrocytes (B), MBP+ myelin (D), NG2+ OPCs (F), and BCAS1+ differentiating oligodendrocytes (H) at baseline, after 4 weeks of cuprizone‐treatment (Cuprizone), and following two (Early) and five (Late) weeks of recovery. Dashed yellow lines denote superficial (Sup, 0–250 μm), middle (Mid, 250–500 μm) and deep (500–750 μm) cortical regions relative to the pial surface. Scale bar, 50 μm. (C, E, G, I) Quantification of the total number of ASPA+ oligodendrocytes (C), myelin area coverage (%) (E), number of NG2+ OPCs (G), and number of BCAS1+ differentiating oligodendrocytes (I) in the deep (500–750 μm) cortical region. Significant comparisons are: (C): Baseline mice (*n* = 4) vs. Cuprizone mice (*n* = 5), *p* = 0.0010; vs. Early Recovery mice (*n* = 5), *p* < 0.0001; and vs. Late Recovery mice (*n* = 6), *p* = 0.0108. (E) Cuprizone mice (*n* = 6) vs. Late Recovery mice (*n* = 6), *p* = 0.0321; Early Recovery mice (*n* = 4) vs. Late Recovery mice (*n* = 6), *p* = 0.0187. (G & I) No significant comparisons. Statistical comparisons are one‐way ANOVA with Tukey correction. Horizontal bar represents mean value; error bars, standard error of the mean. Dots indicate the number of mice per condition. *p < 0.05; ***p < 0.001; ****p < 0.0001.

To establish an immunostaining approach to study barriers to cortical oligodendrocyte replacement, we fed adult mice 0.2% cuprizone‐supplemented chow to induce demyelination and then allowed them to recover from cuprizone supplementation for either 2 (early) or 5 (late) recovery weeks, key time points based on our prior study (Orthmann‐Murphy et al. 2020) (Figure 1A). Previously, we combined in vivo two‐photon imaging with the cuprizone diet to longitudinally monitor the fate of individual oligodendrocytes over the course of demyelination and remyelination (Orthmann‐Murphy et al. 2020). We found that new oligodendrocytes formed after cessation of the cuprizone‐supplemented diet, with a peak rate of formation at 2 weeks post‐cuprizone (“early” recovery). Nearly all oligodendrocytes present at 5 weeks (“late”) recovery were newly formed. Due to the exquisite spatial and temporal resolution of in vivo imaging, we also showed that the loss and formation of oligodendrocytes overlapped in early recovery, a finding that is difficult to appreciate by immunostaining alone.

In the current study, we extended the cuprizone‐administration time to 4 weeks to potentially reduce the overlap of dying and newly forming oligodendrocytes. We then quantified the number of ASPA+ oligodendrocytes after cuprizone treatment (“Cuprizone”), and after early and late recovery, and compared to baseline (Figure 1). We focused on the upper somatosensory cortical region, from the pial surface down to 750 μm, which is a greater cortical depth than is accessible by two‐photon imaging (Figures 1B and S1A). First, we confirmed that, compared to baseline, there are fewer ASPA+ oligodendrocytes after cuprizone supplementation and at early recovery (Figures 1B and S1A). At late recovery, the total number of ASPA+ oligodendrocytes is similar to baseline (Figures 1B and S1A). Notably, this current approach does not detect subtle changes in oligodendrocyte density within this full (500 μm × 750 μm) region. In our prior in vivo imaging study, however, we showed that deep cortical oligodendrogenesis was selectively impaired, with only ~55% of the baseline oligodendrocyte population replaced after 9 weeks of recovery in the deepest cortical zone examined (200–300 μm; Orthmann‐Murphy et al. 2020).

To determine whether we could detect impaired deep cortical oligodendrocyte replacement with our current approach, we compared the number of ASPA+ oligodendrocytes in superficial (relative to pial surface, 0–250 μm; Sup), middle (250–500 μm; Mid), and deep (500–750 μm; Deep) cortical regions at each time point (Figures 1C and S1A). In the deep cortical region, there were fewer ASPA+ oligodendrocytes after cuprizone, and at early and late recovery compared to baseline (Figure 1C). In the middle cortical region, there were also significantly fewer ASPA+ oligodendrocytes at early recovery compared to baseline (Figure S1A). We did not detect significant changes in the number of superficial ASPA+ oligodendrocytes at any time point (Figure S1A), despite previously showing that superficial oligodendrocytes are fully replaced by 5 weeks of recovery (Orthmann‐Murphy et al. 2020). Therefore, with this current approach leveraging key recovery time points, we can detect incomplete oligodendrocyte replacement in deep (500–750 μm) cortex by late recovery, and so we will focus the remainder of our study on this deep cortical region.

To confirm that cortical myelin density follows similar dynamics as ASPA+ oligodendrocytes over this time course, we quantified the percentage of myelin basic protein (MBP) signal within these cortical regions (Figure 1D,E). In the deep cortical region, MBP signal becomes patchy at early recovery (Figure 1D). We suspect these patches represent the loss of the cohort of myelin sheaths belonging to individual ASPA+ oligodendrocytes, as each cell can form ~50 myelin sheaths (Orthmann‐Murphy et al. 2020). Overall, the dynamics of myelin area coverage following cuprizone treatment and through recovery is similar to ASPA+ oligodendrocytes.

One potential barrier to cortical oligodendrocyte replacement is the depletion of OPCs, or the precursor pool available to form new oligodendrocytes. To address the possibility that cuprizone administration depletes the OPC pool, we quantified the number of cortical NG2+ OPCs at each post‐cuprizone and recovery time point (Figures 1F,G and S1C). Similar to our prior study (Orthmann‐Murphy et al. 2020), we found no significant changes in OPC number in the deep cortex (Figure 1G, also see Figure S1C).

Given there are fewer oligodendrocytes at late recovery, but no changes in OPC number in the deep cortical region, it is possible that cuprizone administration is toxic to an intermediary stage of oligodendrogenesis. Recent studies showed that BCAS1 (breast carcinoma amplified sequence 1) is expressed by a subset of the oligodendrocyte lineage: newly differentiated, pre‐myelinating, and early‐myelinating oligodendrocytes that derive from NG2+ OPCs but do not yet express all mature oligodendrocyte markers (Chapman et al. 2024; Fard et al. 2017). Following oligodendrocyte loss, we expected to find higher numbers of BCAS1+ oligodendrocytes during recovery, particularly at early recovery when there is peak formation of new oligodendrocytes (Orthmann‐Murphy et al. 2020). Instead, we found that the number of deep cortical BCAS1+ oligodendrocytes was similar to baseline at all time points (Figures 1H,I and S1D). This lack of compensatory increase in BCAS1+ differentiating oligodendrocytes in the setting of maintained OPC density supports the hypothesis that there are external barriers to forming replacement oligodendrocytes in the deep cortex following cuprizone administration.

### 3.2 Cortical Microglia Adopt Spatially Heterogeneous Reactive States Following Demyelination

Both microglia and astrocytes contribute to cuprizone‐induced pathology and could influence oligodendrocyte regeneration (Vega‐Riquer et al. 2019). We previously showed that deep cortical astrocytes persistently upregulate GFAP following cuprizone administration (Orthmann‐Murphy et al. 2020); the reactive responses of cortical microglia to cuprizone‐induced demyelination are not yet characterized. To do so, we quantified the number of cortical Iba1+ cells (which should mainly represent microglia (Zhao et al. 2023)) in Mobp‐eGFP mice (or their wild‐type littermates) following cuprizone administration and at early and late recovery. Compared to baseline, after cuprizone we observed a 47.85% ± 6.46% increase in the number of Iba1+ cells throughout the full cortical region, with a persistent elevation through early recovery (89.7 ± 2.21 cells Baseline mice (*n* = 5) vs. 132.6 ± 17.62 cells Cuprizone mice (*n* = 4), *p* = 0.0305; vs. 134.0 ± 8.5 cells Early Recovery mice (*n* = 7), *p* = 0.0073). Iba1+ cell numbers approached baseline levels by late recovery (89.7 ± 2.21 cells Baseline mice (*n* = 5) vs. 105.1 ± 5.1 cells Late Recovery mice (*n* = 5), *p* = 0.8067). This observed Iba1+ cell increase after cuprizone and through early recovery was primarily due to higher numbers of Iba1+ microglia in the deep cortical region (31.4 ± 1.8 cells Baseline mice (*n* = 5) vs. 67.6 ± 9.6 cells Cuprizone mice (*n* = 4), *p* = 0.0014; vs. 73.89 ± 3.3 cells Early Recovery mice (*n* = 7), *p* = 0.0001).

To better characterize the reactive states of cortical microglia following cuprizone‐induced demyelination, we quantified changes in morphology. To do so, we first defined cortical microglia as either Iba1+ cells in wild‐type (WT; includes MOBP‐egfp mice or WT littermate controls), or GFP+ cells in Cx3Cr1‐eGFP transgenic mice. In Cx3Cr1‐eGFP mice, GFP is expressed by CNS‐resident macrophages (Jung et al. 2000), which, as noted above, are predominantly represented by microglia (Zhao et al. 2023). To ensure that these transgenic lines could be used interchangeably to study cortical microglia reactivity to cuprizone, we confirmed that ASPA+ oligodendrocyte dynamics following cuprizone administration in Cx3Cr1‐eGFP mice were similar to WT mice (Mobp‐eGFP and WT littermate controls; Figure S1E,F). Then, we quantified and compared the number of Iba1+ cells in WT to GFP+ cells in Cx3Cr1‐eGFP mice and found no significant differences (Figure S2E). Last, nearly all Iba1+ cells in Cx3cr1‐eGFP are also GFP+ (Figure S2F). Together, this data supports using both WT and Cx3Cr1‐eGFP mice to characterize the reactive states of cortical microglia following cuprizone.

**FIGURE 2:**
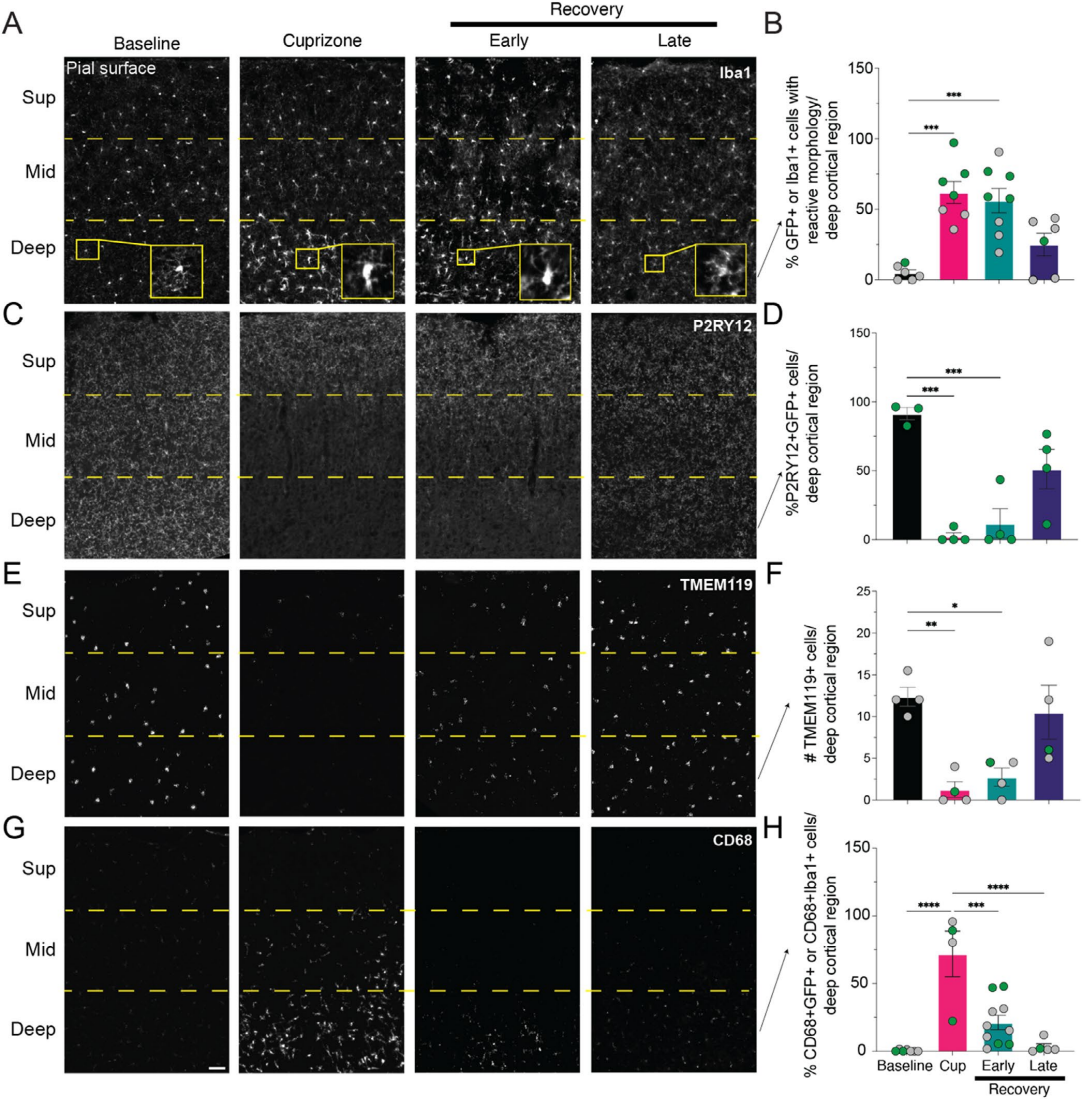
Spatial and temporal heterogeneity of cortical microglia reactive responses to demyelination. (A) Representative coronal images from Mobp‐eGFP or WT littermate mice at baseline, after 4 weeks cuprizone‐treatment (Cuprizone) and following two (Early) or five (Late) weeks of recovery immunostained for Iba1. Insets show representative images of microglia cell morphology in the deep cortical region. (C, E, G) Representative coronal images from Mopb‐eGFP, Cx3Cr1‐eGFP mice or WT littermates at baseline, after 4 weeks of cuprizone‐treatment (Cuprizone) and following two (Early) or five (Late) weeks of recovery. Sections were immunostained for GFP in Cx3Cr1‐eGFP mice or Iba1 in Mobp–eGFP or WT littermates (not shown) and co‐labeled with P2RY12 (C) or CD68 (G). (E) In situ RNA labeling for Tmem119. Dashed yellow lines denote superficial (Sup, 0–250 μm), middle (Mid, 250–500 μm) and deep (500–750 μm) cortical regions relative to the pial surface. Scale bar, 50 μm. (B, D, F, H) Quantification in deep (500–750 μm) cortical regions of (B) the proportion of Iba1+ or GFP+ cells with reactive morphology, (D) the proportion of GFP+ cells that were also P2RY12+, (F) the number of TMEM119+ cells, and (H) the proportion of Iba1+ or GFP+ cells that were also CD68+. Significant comparisons are: (B) Baseline mice (*n* = 6) vs. Cuprizone mice (*n* = 7), *p* = 0.0001; vs. Early Recovery mice (*n* = 8), *p* = 0.0004. (D) Baseline mice (*n* = 3) vs. Cuprizone mice (*n* = 4), *p* = 0.0004; vs. Early Recovery mice (*n* = 4), *p* = 0.0009. (F) Baseline mice (*n* = 4) vs. Cuprizone mice (*n* = 4), *p* = 0.0055; vs. Early Recovery mice (*n* = 4), *p* = 0.0149. (H) Baseline mice (*n* = 7) vs. Cuprizone mice (*n* = 4), *p* < 0.0001; Cuprizone mice (*n* = 4) vs. Early Recovery mice (*n* = 10), *p* = 0.0002; vs. Late Recovery mice (*n* = 5), *p* < 0.0001. Statistical comparisons are one‐way ANOVA with Tukey correction. Horizontal bar represents mean value; error bars, standard error of the mean. Dots indicate the number of mice per condition; green dots are Cx3Cr1–eGFP mice and gray dots are WT mice (Mobp‐eGFP or WT littermate controls). *p < 0.05; **p < 0.01; ***p < 0.001; ****p < 0.0001.

Homeostatic microglia typically have a small circular cell body and ramified processes, whereas in the setting of injury, reactive microglia typically have larger cell bodies with an amoeboid shape and short, thick processes (Woodburn et al. 2021). Using these criteria, we quantified the number of cortical microglia with reactive morphology at each time‐point (Figure 2A,B, insets in A). Reactive microglia morphology appeared in deep cortical regions after 4 weeks of cuprizone (Figure 2A,B) and persisted at early recovery. Homeostatic morphology predominated by late recovery in deep cortex. Contrary to deep cortex, most superficial cortical microglia did not adopt a reactive morphology at any time‐point, but middle region cortical microglia adopted a reactive morphology in a similar temporal pattern as the deep cortical microglia (Figure S2A).

To further characterize cortical reactive microglia functional changes following demyelination, we immunostained for both homeostatic and reactive markers of microglia in either WT (as defined above) or Cx3Cr1‐eGFP transgenic mice fed the cuprizone diet. To determine changes in homeostatic marker P2RY12 expression (Haynes et al. 2006), we co‐immunostained for both P2RY12 and GFP in Cx3Cr1‐eGFP mice from each time point. After 4 weeks of cuprizone, deep cortical GFP+ microglia downregulated the expression of P2RY12, and this persisted through early recovery (Figure 2C,D). This pattern was similarly observed in middle cortical microglia after 4 weeks of cuprizone and through early recovery, and P2RY12 expression returned to baseline levels by late recovery (Figure S2B). Superficial cortical microglia, by comparison, did not decrease P2RY12 expression following cuprizone treatment (Figure S2B). In parallel, to ensure our findings were specific to microglia, we performed in situ RNA labeling for TMEM119, a canonical microglia marker not expressed by other CNS resident macrophages (Bennett et al. 2016). Similar to P2RY12, there were fewer TMEM119+ cells in the deep cortex after 4 weeks of cuprizone and through early recovery (Figure 2E,F), but not at late recovery. Superficial cortical microglia did not significantly reduce TMEM119 expression after cuprizone administration (Figure S2C).

In addition to morphology changes, reactive microglia are commonly identified by upregulation of CD68, a lysosomal protein expressed by activated and phagocytosing microglia and macrophages (Bennett and Viaene 2021). We found that deep cortical microglia upregulate CD68 after 4 weeks of cuprizone, and only some deep cortical microglia maintained their expression in early recovery (Figure 2G), but the comparison to baseline was not significant (Figure 2H). By late recovery, CD68 expression decreased and appeared similar to baseline across all cortical depths. In contrast, superficial cortical microglia did not upregulate CD68 at any time‐point, while middle cortical region microglia followed a similar temporal CD68 expression pattern as deep cortical microglia (Figure S2D).

Overall, we observed a spatially heterogeneous cortical reactive microglia response to demyelination, where deep cortical microglia changed morphology, downregulated homeostatic markers P2RY12 and TMEM119, and upregulated CD68, and this response persisted through early recovery. In contrast, superficial microglia retained homeostatic morphology and marker expression similar to that observed at baseline. With these observations, and our finding that deep cortical ASPA+ oligodendrocytes are incompletely replaced (Figure 1), we hypothesized that demyelination induces deep cortical microglia to adopt a functional reactive phenotype that impairs the formation of new oligodendrocytes.

### 3.3 Depleting Cortical Reactive Microglia During Recovery Restores Cortical Oligodendrocyte Numbers

**FIGURE 3:**
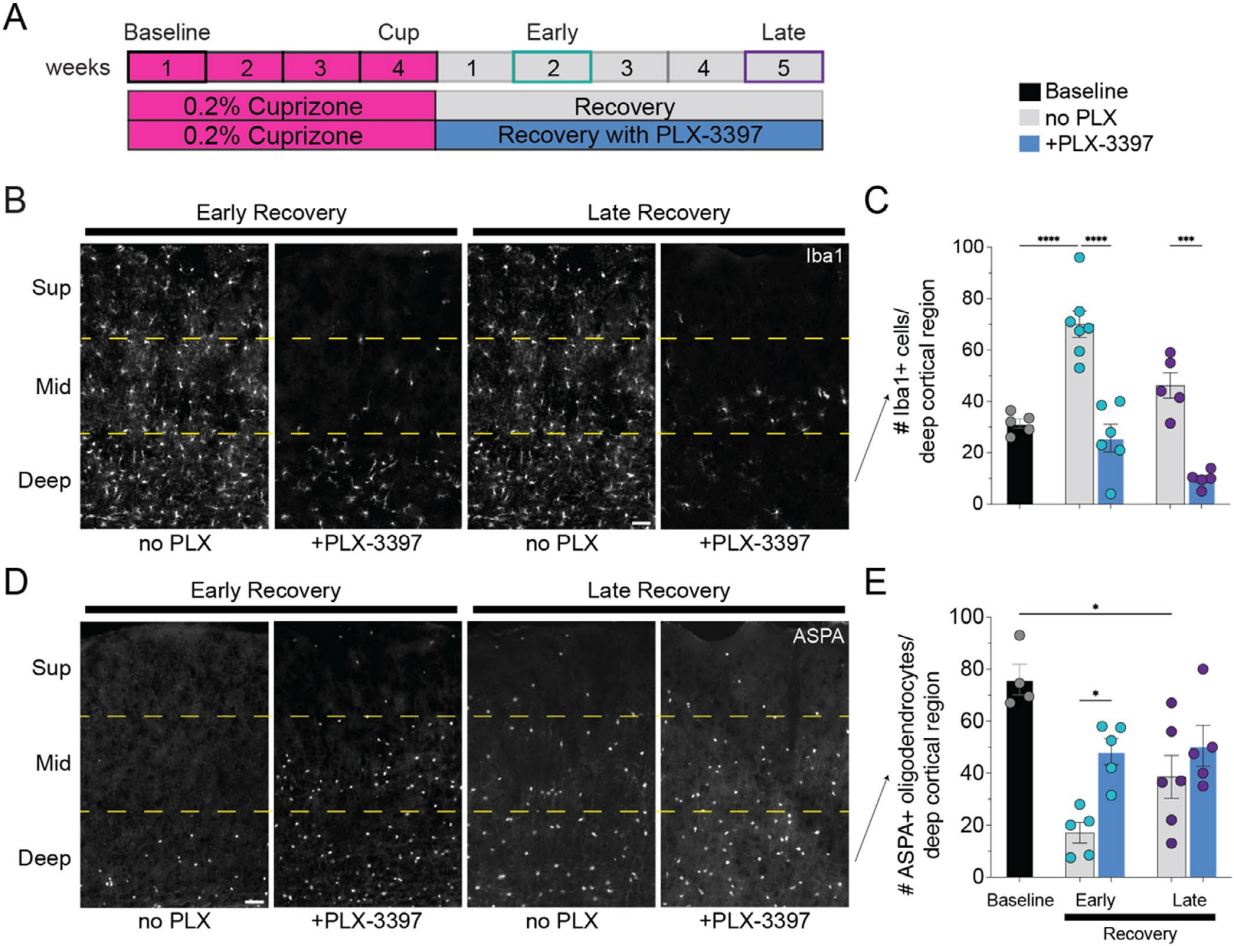
Depletion of cortical reactive microglia restores oligodendrocyte numbers during early recovery. (A) Experimental paradigm with administration of Csf1r antagonist PLX‐3977 post‐cuprizone to deplete reactive microglia during recovery. (B, D) Representative coronal images depicting Iba1+ cortical microglia (B) and ASPA+ oligodendrocytes (D) at early and late recovery without PLX‐3977 (no PLX, left) and with PLX‐3977 administration (right). Dashed yellow lines denote superficial, middle and deep cortical regions. Scale bar, 50 μm. (C) (E) Quantification of the number of Iba1+ microglia (C) and ASPA + oligodendrocytes (E) in the deep (500–750 μm) somatosensory cortical region in mice treated with cuprizone followed by recovery (gray) or treatment with PLX‐3397 (blue). Significant comparisons are: (C) Baseline mice (*n* = 5) vs. Early Recovery mice (*n* = 7), *p* < 0.0001; Early Recovery No PLX mice (*n* = 7) vs. Early Recovery + PLX‐3397 mice (*n* = 6), *p* < 0.0001; Late Recovery No PLX mice (*n* = 5) vs. Late Recovery + PLX‐3397 mice (*n* = 5), *p* = 0.0003. (E) Early Recovery no PLX mice (*n* = 5) vs. Early Recovery + PLX‐3397 mice (*n* = 5), *p* = 0.0391; Baseline mice (*n* = 4) vs. Late Recovery no PLX mice (*n* = 6), *p* = 0.0108 (also shown in Figure 1C). Statistical comparisons are one‐way ANOVA with Tukey correction. Horizontal bar represents mean value; error bars, standard error of the mean. Dots indicate the number of mice per condition; teal dots represent early recovery and purple dots represent late recovery. *p < 0.05; ***p < 0.001; ****p < 0.0001.

To determine whether deep cortical reactive microglia impair oligodendrocyte regeneration, we fed WT (Mobp‐eGFP or WT littermates) mice chow supplemented with Csf1r inhibitor PLX‐3397 (Elmore et al. 2014) to deplete microglia following cuprizone administration (Figure 3A). To confirm microglia depletion post‐cuprizone, we administered PLX‐3397 for 2 or 5 weeks following cuprizone‐supplemented chow and quantified the number of Iba1+ cells in the full somatosensory cortex region, pial surface down to 750 μm (Figure 3B). After 2 weeks, there were substantially fewer Iba1+ cortical microglia, but they were not fully eliminated. In comparison to baseline, Iba1+ cells in the superficial region were reduced by 90.64% ± 37.32% at early recovery, and then by 90.22% ± 41.82% at late recovery (Superficial region, Baseline mice (*n* = 5) vs. Early Recovery + PLX‐3397 mice (*n* = 6), *p* < 0.0001; vs. Late Recovery + PLX‐3397 mice (*n* = 5), *p* < 0.0001). In the deep cortical region, only 17.9% ± 3.91% of cortical microglia were depleted compared to baseline at early recovery, and then further reduced to 68.8% ± 10.84% of baseline by late recovery (Figure 3C). However, we also found that the number of Iba1+ cells increased significantly in response to cuprizone and remained elevated through early recovery (Figure 3C, gray bars). If we compare instead to the number of Iba1+ cells at each recovery time point following cuprizone administration, treatment with PLX‐3397 led to 63.25% ± 14.05% and then 78.79% ± 14.28% fewer Iba1+ cells at early and late recovery, respectively, compared to recovery conditions without PLX‐3397 (Figure 3C).

While our findings are consistent with previous studies that showed incomplete depletion of microglia with PLX‐3397 (Najafi et al. 2018), we also tested whether administration of a different Csf1r inhibitor, PLX‐5622, would show improved Iba1+ cell depletion over PLX‐3397. PLX‐5622 has 100‐fold higher selectivity for Csf1r compared to other members of the platelet‐derived growth factor receptor (PDGFRα) family of type III receptor tyrosine kinases (Liu et al. 2019). Despite this selectivity difference, administration of PLX‐5622 following cuprizone administration during recovery resulted in similar levels of microglial depletion compared to PLX‐3397 (Figure S3B).

To determine the effect of reactive microglia depletion on cortical oligodendrocyte regeneration, we quantified the number of ASPA+ oligodendrocytes at early and late recovery in the presence of Csf1r inhibitor treatment. There were ~3‐fold more ASPA+ oligodendrocytes at early recovery in the full cortical region with Csf1r inhibitor treatment, compared to no PLX treatment (30.9 ± 6.21 ASPA+ cells Early Recovery no PLX (*n* = 5) vs. 90.5 ± 11.66 ASPA+ cells Early Recovery + PLX‐3397 mice (*n* = 5), *p* = 0.0328), and this effect was primarily due to higher numbers of ASPA+ oligodendrocytes in the deep cortical region (Figure 3E). In fact, we found that the number of ASPA+ oligodendrocytes in the deep cortex was equivalent to baseline oligodendrocyte density at early recovery, and this persisted through late recovery (Figure 3E). In contrast, in the no PLX condition, the number of ASPA+ oligodendrocytes at late recovery was significantly reduced compared to baseline (Figure 1C). This finding in PLX‐treated mice suggests that more mature ASPA+ oligodendrocytes were formed by late recovery, approaching baseline levels. We did not detect significant changes in myelin density in mice treated with PLX‐3397, although the MBP+ signal was less patchy in some examples of PLX‐treated mice at early recovery (Figure S3E).

If our observations were due to off‐target effects of PLX‐3397 on PDGFRα expressed by OPCs, we would expect that treatment with PLX‐5622 post‐cuprizone could result in relatively higher numbers of OPCs and possibly more ASPA+ oligodendrocytes at early recovery, compared to PLX‐3397. However, we found that NG2+ OPC density was maintained in the deep cortical region during early recovery when treated with either Csf1r inhibitor (Figure S3D). Unlike treatment with PLX‐3977, PLX‐5622 treatment during recovery did not lead to significantly higher numbers of ASPA+ oligodendrocytes at early recovery compared to non‐PLX mice (Figure S3C). However, late recovery ASPA+ oligodendrocyte numbers in PLX‐5622 treated mice were not significantly different from baseline, suggesting ASPA+ cell numbers could be following a similar trajectory as PLX‐3397 treated mice over the course of recovery. Overall, the effect of PLX‐5622 on oligodendrocyte recovery was less robust than PLX‐3397.

**FIGURE 4:**
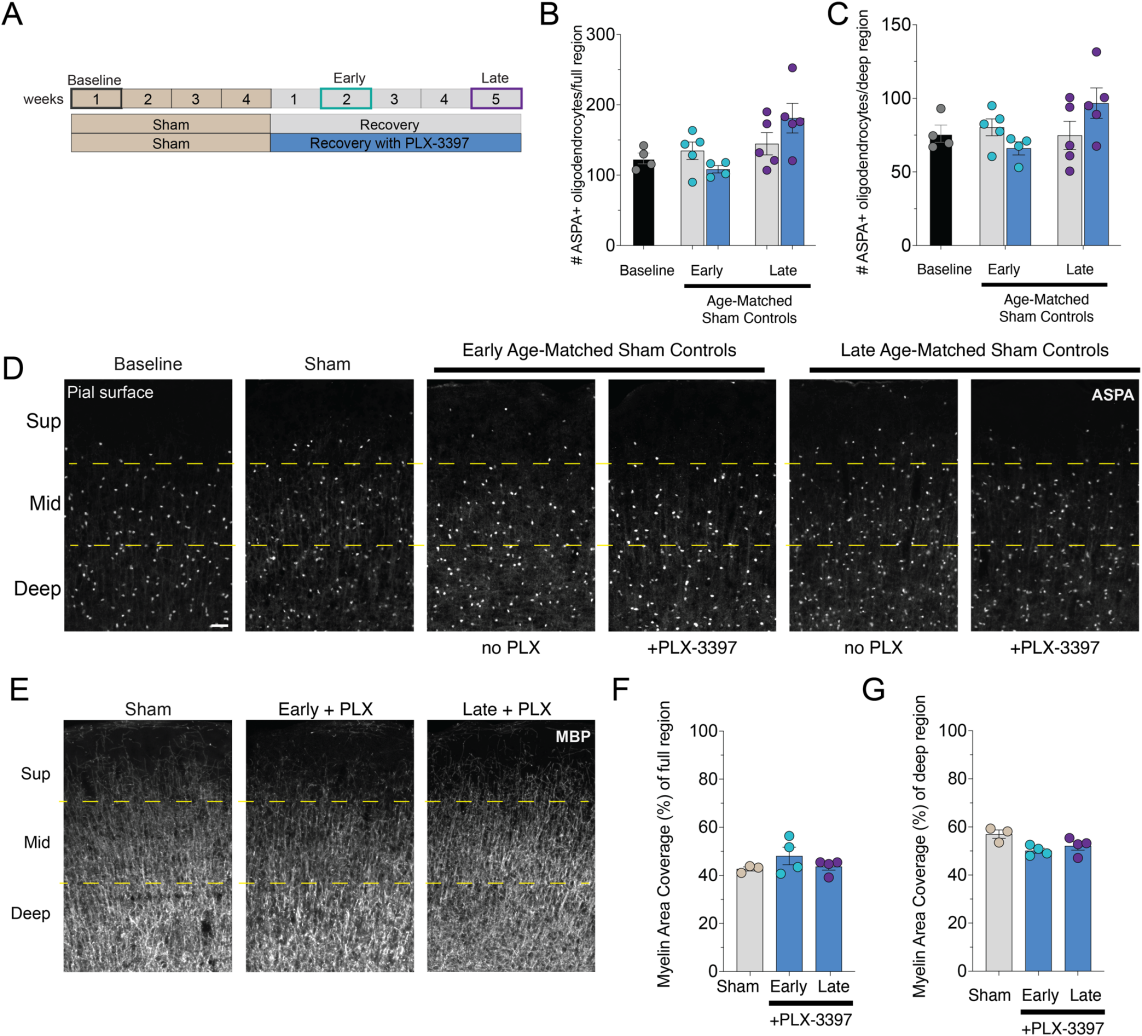
Cortical oligodendrocyte density is stable with or without microglia in the healthy brain. (A) Experimental schematic. (B, C) Quantification of ASPA+ oligodendrocytes in healthy adult mice at baseline, and after 2 and 5 weeks following 4 weeks of sham‐administration without (gray) or with PLX‐3397 treatment (blue) in the full cortical region (B) or deep region (C). Representative coronal images of ASPA+ oligodendrocytes (D) and MBP+ myelin (E) at baseline, after 4 weeks of sham diet, then after 2 and 5 weeks post‐sham either without (no PLX) or with PLX‐3397 (+PLX‐3397) treatment. Dashed yellow lines denote superficial (Sup, 0–250 μm), middle (Mid, 250–500 μm) and deep (500–750 μm) cortical regions relative to the pial surface. Scale bar, 50 μm. (F, G) Quantification of the myelin area coverage based on MBP signal in healthy adult mice at baseline, and after 2 and 5 weeks following 4 weeks of sham‐administration without (gray) or with PLX‐3397treatment (blue) in the full cortical region (F) or deep region (G). Statistical comparisons are one‐way ANOVA with Tukey correction, with no significant comparisons. Horizontal bar represents mean value; error bars, standard error of the mean. Dots indicate the number of mice per condition; teal dots represent early recovery and purple dots represent late recovery.

To test whether the effect of post‐cuprizone PLX‐3397 treatment on oligodendrocyte density was specific to “reactive” cortical microglia, we administered PLX‐3397 to adult mice following administration of sham feed (Figure 4). We found that treatment with 2 weeks of PLX‐3397 post‐sham treatment depleted the majority of Iba1+ cells compared to baseline in both the full (89.7 ± 2.21 cells Baseline mice (*n* = 5) vs. 15.17 ± 5.86 cells 2 weeks post‐sham + PLX‐3397 mice (*n* = 6), *p* < 0.0001) and the deep region (31.4 ± 1.81 cells Baseline mice (*n* = 5) vs. 5.0 ± 1.83 cells 2 weeks post‐sham + PLX‐3397 mice (*n* = 6), *p* < 0.0001). Despite significant microglia depletion, ASPA+ cortical oligodendrocyte numbers were not significantly changed after 2 or 5 weeks of PLX‐3397 treatment in the sham‐treated cortex (Figure 4B–D). MBP+ myelin area coverage of these sham‐treated cortical regions was also not altered after 2 or 5 weeks of PLX‐3397 treatment compared to sham alone (Figure 4E–G). This finding highlights that deep cortical reactive microglia adopt novel functions post‐cuprizone that influence oligodendrocyte density.

Given that microglia and astrocytes influence each other's state and function (Matejuk and Ransohoff 2020), we wanted to ensure that cortical astrocyte numbers were not also altered by microglia depletion following demyelination. To determine cortical astrocyte density, we quantified the number of GFAP+ astrocytes after 2 and 5 weeks of recovery during PLX‐3397 administration. We found that the number of cortical reactive astrocytes was unchanged by microglia depletion (Figure S5). Similar to our previous findings (Orthmann‐Murphy et al. 2020), even in the presence of Csf1r inhibitor, deep cortical reactive astrocytes persistently upregulate GFAP (Figure S5B).

### 3.4 Cortical Reactive Microglia Impair the Generation of Differentiating Oligodendrocytes

**FIGURE 5:**
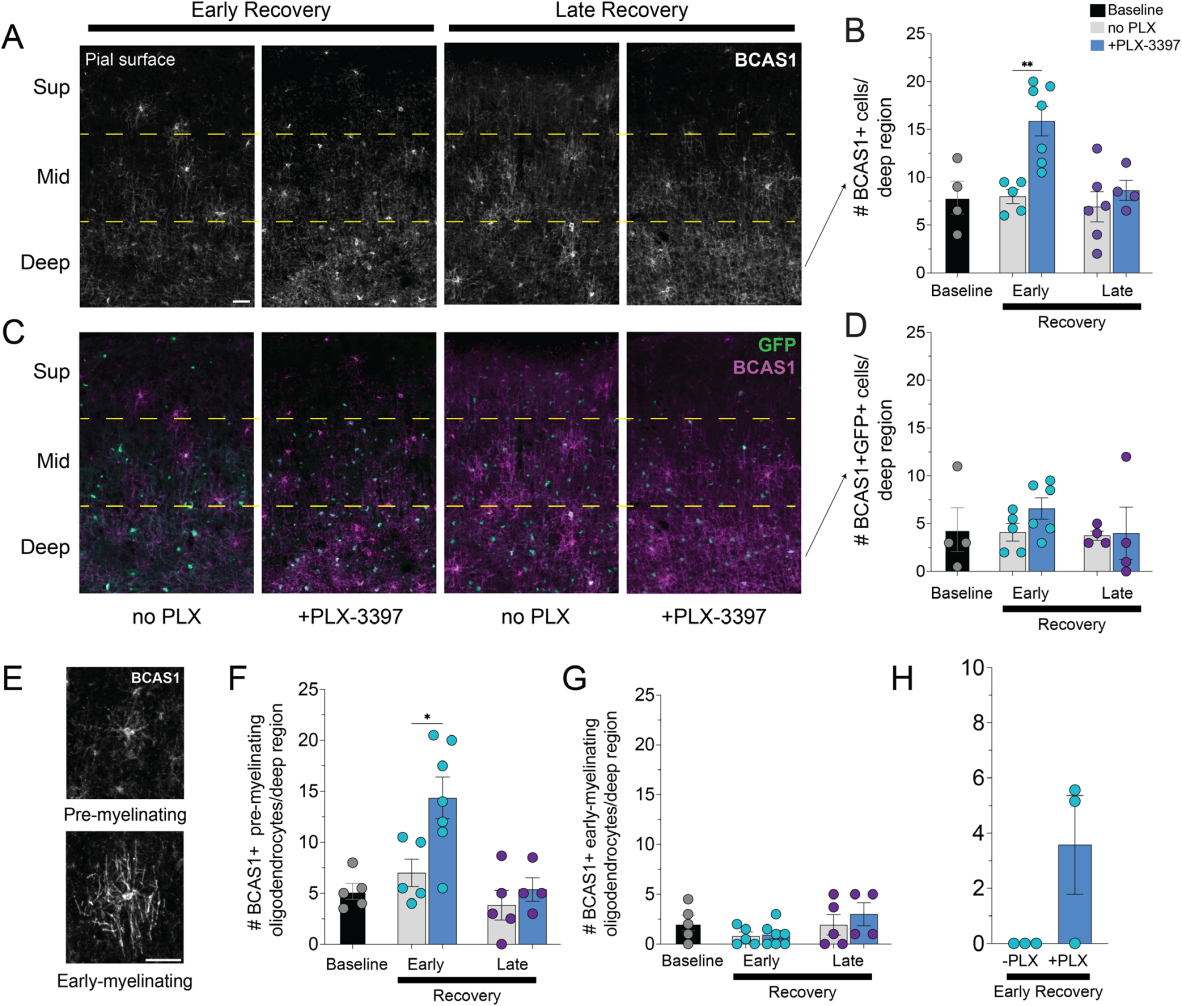
Depletion of cortical reactive microglia promotes the formation of differentiating oligodendrocytes during early recovery. (A, C, E) Representative images of coronal images immunolabeled for BCAS1+ cells (gray, A), both GFP+ (green) and BCAS1+ (magenta) cells in Mobp–eGFP mice (C), at early and late recovery, without (no PLX) or with PLX‐3397, and representative BCAS1+ cells with pre‐myelinating and early‐myelinating morphology (E). Dashed yellow lines denote superficial (Sup, 0–250 μm), middle (Mid, 250–500 μm) and deep (500–750 μm) cortical regions relative to the pial surface. Scale bar, 50 μm. (B, D, F, G) Quantification of the number of BCAS1+ cells (B), BCAS1 + GFP+ cells (D), pre‐myelinating BCAS1+ cells (F) and early‐myelinating BCAS1+ cells (G) in the deep (500–750 μm) cortical region cortex at baseline, and early and late recovery without (gray) or with PLX‐3397 (blue). (H) Quantification of the proportion of deep cortical BCAS1+ cells that are CC3+ at early recovery without (‐PLX) or with PLX‐3397. Significant comparisons are (B) Early Recovery no PLX mice (*n* = 5) vs. Early Recovery + PLX‐3397 mice (*n* = 7), *p* = 0.0040. (F) Early Recovery no PLX mice (*n* = 5) vs. Early Recovery + PLX‐3397 mice (*n* = 7), *p* = 0.0163. Statistical comparisons are one‐way ANOVA with Tukey correction. Horizontal bar represents mean value; error bars, standard error of the mean. Dots indicate number of mice per condition; teal dots represent early recovery and purple dots represent late recovery. *p < 0.05; **p < 0.01.

There are several possibilities to explain how depleting cortical microglia post‐cuprizone‐induced demyelination restores the deep cortical ASPA+ oligodendrocyte population at early and late recovery. The first is that there is delayed clearance of dying baseline oligodendrocytes, as deep cortical reactive microglia are not present to phagocytose these cells (Olveda et al. 2024). Another possibility is that there are more differentiating and newly forming oligodendrocytes at early recovery in the absence of reactive microglia. To test the latter possibility, we quantified the number of BCAS1+ newly formed oligodendrocytes (Fard et al. 2017) in the early and late recovery period in mice treated with PLX‐3397 (Figure 5). At early recovery with PLX‐3397, there were more cortical BCAS1+ oligodendrocytes in the full cortical region compared to no PLX treatment (17.6 ± 2.03 BCAS1+ cells Early Recovery no PLX mice (*n* = 5) vs. 30.5 ± 1.68 BCAS1+ cells Early Recovery + PLX‐3397 mice (*n* = 7), *p* = 0.0203), with ~2‐fold more BCAS1+ cells in the PLX‐treated deep cortical region (Figure 5A,B).

Notably, both pre‐myelinating and early‐myelinating oligodendrocytes express BCAS1 (Fard et al. 2017). To determine whether there were more early‐myelinating BCAS1+ oligodendrocytes in the absence of cortical reactive microglia, we co‐labeled for BCAS1 and GFP in Mobp‐eGFP mice treated with cuprizone followed by recovery alone or treatment with PLX‐3397. BCAS1 + GFP+ oligodendrocytes should represent a relatively more mature differentiating oligodendrocyte state than BCAS1 + GFP‐ cells (Chapman et al. 2024; Hughes et al. 2018; Orthmann‐Murphy et al. 2020). We saw no difference in the number of BCAS1 + GFP+ cells at baseline, after 4 weeks of cuprizone, or after recovery with or without PLX‐3397 (Figure 5C,D), despite the restored baseline levels of deep cortical ASPA+ oligodendrocytes by late recovery with PLX‐treatment (Figure 3). We also quantified BCAS1+ cells by morphology, defining pre‐myelinating cells and early‐myelinating BCAS1+ cells as previously described (Figure 4E; corresponding to resting and activated BCAS1+ cells, respectively, from Bergner et al. 2024). There were more pre‐myelinating BCAS1+ cells at early recovery in the PLX‐treated mice (Figure 5F) but no difference in the number of early‐myelinating BCAS1+ cells at any time point (Figure 5G). These findings demonstrate that depleting microglia after cuprizone treatment leads to higher numbers of deep cortical pre‐myelinating BCAS1+ oligodendrocytes, thereby forming a larger available pool of differentiating oligodendrocytes during recovery from demyelination.

Could this larger pool of pre‐myelinating BCAS1+ cells in early recovery (formed when microglia were depleted) form mature myelinating oligodendrocytes? We previously showed in the healthy middle‐aged cortex that only 22% of pre‐myelinating oligodendrocytes stably integrated into the cortex (Hughes et al. 2018), and the remaining differentiating cells died. Most of these differentiating cells likely died by apoptosis; BCAS1+ cells were recently shown to die by a caspase‐3 dependent mechanism (Chapman et al. 2024). To determine whether this larger pool of pre‐myelinating BCAS1+ cells in PLX‐treated mice was at increased risk of cell death, we quantified the number of BCAS1+ oligodendrocytes that expressed cleaved caspase 3 (CC3; (Chapman et al. 2024)), during early recovery, with or without PLX treatment (Figure 5H). Only a small proportion of deep cortical BCAS1+ cells (all with pre‐myelinating morphology, not shown) expressed CC3 (3.57% ± 1.79), corresponding to a ~1 BCAS1+ cell per region quantified. Although we would not expect all BCAS1+ cells to survive based on prior studies, the majority of BCAS1+ cells appear to be healthy at early recovery in PLX‐treated mice and could mature into ASPA+ oligodendrocytes. Therefore, depletion of deep cortical *reactive* microglia (as characterized in Figures 2 and S2) after cuprizone‐induced demyelination increases the pool of differentiating oligodendrocytes, and these cells are likely responsible for restoring baseline numbers of deep cortical ASPA+ oligodendrocytes by late recovery (Figure 3). Together, our findings indicate that deep cortical microglia adopt a reactive state in response to demyelination that reduces oligodendrocyte numbers during early recovery.

## 4 Discussion

There are currently no available reparative treatments to fix demyelinated lesions in multiple sclerosis (MS). The long‐term consequence of demyelination in the CNS is axonal damage and neurodegeneration (Chang et al. 2002) in both white and gray matter lesions, leading to progressive disease. Progressive forms of MS manifest with gait and cognitive impairment, correlating to brain and spinal atrophy (Reich et al. 2018). Although OPCs represent an available pool to replace lost oligodendrocytes and myelin, replacement is limited, particularly in cortical lesions (Chang et al. 2012). Microglia are a salient feature of white and gray matter lesions, representing a major part of ongoing compartmentalized inflammation induced by an initial inflammatory attack (Absinta et al. 2021). Accumulating evidence shows that microglia clear myelin debris and influence oligodendrocytes and myelin formation in MS lesions (Irfan et al. 2022; Li and Barres 2018). Although there is a growing understanding of the diverse reactive states and functions that microglia adopt in response to CNS damage (Tan et al. 2020), prior to this study, it was not known whether cortical microglia promote or impair oligodendrocyte recovery after cortical demyelination.

Here, we determined whether cortical reactive microglia are a barrier to oligodendrocyte regeneration following cuprizone‐induced injury. To do so, we adapted our previously reported platform, which combined cuprizone treatment and high‐resolution longitudinal in vivo imaging to demonstrate that deep cortical oligodendrocyte regeneration is impaired (Orthmann‐Murphy et al. 2020). Leveraging the key time points defined in this prior study, we performed immunostaining and in situ RNA labeling to determine the temporal and spatial heterogeneity of cortical reactive microglia states following cuprizone treatment, and then determined how these cortical reactive microglia influence oligodendrocyte recovery. We extended the cuprizone treatment time course to allow more time for baseline oligodendrocyte degeneration, 5 and quantified oligodendrocyte recovery after 2 and 5 weeks post‐cuprizone. In our prior study, 2 weeks, or “early” recovery, corresponded to the time with the highest rate of new oligodendrocyte formation. “Late” recovery, or 5 weeks post‐cuprizone, reflected the time point when nearly all cortical oligodendrocytes were newly formed (Orthmann‐Murphy et al. 2020).

By quantifying ASPA+ oligodendrocytes with this updated approach, we confirmed that the mature oligodendrocyte population is reduced after 4 weeks of cuprizone compared to baseline, reaches a nadir at early recovery, and then approaches baseline density by late recovery in the upper somatosensory cortex. Similar to our prior study, we found that deep cortical ASPA+ oligodendrocytes were not fully restored to baseline levels at late recovery (Figure 1). Reassuringly, we found that OPC density was maintained at all time points and cortical depths. Given that the OPC pool was maintained, we expected to find an increased number of newly formed BCAS1+ oligodendrocytes at early recovery to account for the increased rate of oligodendrocyte formation we previously showed in Mobp‐eGFP transgenic mice at this time point (Orthmann‐Murphy et al. 2020). Instead, we found no corresponding change in the number of BCAS1+ oligodendrocytes at early or late recovery to match the loss of baseline oligodendrocytes from cuprizone treatment. The combination of limited BCAS1+ differentiating oligodendrocyte formation at early recovery and impaired ASPA+ mature oligodendrocyte replacement at late recovery provided the unique opportunity to study the barriers to oligodendrocyte regeneration with both spatial and temporal resolution.

We then characterized the cortical microglia response to cuprizone‐induced demyelination. We found that deep cortical microglia adopt a reactive state, characterized by an amoeboid morphology, downregulation of homeostatic markers P2RY12 and TMEM119, and upregulation of CD68; this response persists through early recovery and resolves by late recovery. In contrast, superficial microglia maintain homeostatic morphology and expression of homeostatic markers P2RY12 and TMEM119. Taken together, our results reveal spatial and temporal heterogeneity of cortical microglia responses to demyelination. The differential spatial responses of cortical microglia are likely driven, at least in part, by environmental differences. For example, there are more oligodendrocytes in the deep cortex compared to the superficial cortex (Hughes et al. 2018). Following cuprizone‐induced demyelination, deep cortical microglia will be exposed to relatively more myelin debris than superficial microglia, potentially inducing different reactive responses. In addition, deep cortical astrocytes also become persistently reactive (Figure S4; Orthmann‐Murphy et al. 2020) and likely influence deep cortical reactive microglia function (Liddelow and Barres 2017). Whether baseline heterogeneity in cortical microglia (Hammond et al. 2019; Paolicelli et al. 2022; Tarozzo et al. 2003) also influences spatially distinct reactive responses is not yet known. Future studies will further define the heterogeneity and distribution of cortical reactive states in individual microglia, including whether they adopt disease‐associated states previously found in white matter lesions (Miron et al. 2013), other CNS injury models (Paolicelli et al. 2022), or potentially novel reactive microglia states.

We tested whether reactive cortical microglia influence oligodendrocyte regeneration by administering a Csf1r inhibitor (PLX) to deplete microglia during the recovery period. Although administration of PLX depleted microglia throughout the cortical region, we observed more deep cortical ASPA+ oligodendrocytes and BCAS1+ differentiating oligodendrocytes at early recovery (Figures 3 and 5), in the same region and same time period we identified the transient deep cortical reactive microglia phenotype (Figures 2 and S2). The increased number of pre‐myelinating BCAS1+ cells‐oligodendrocyte lineage cells that are predominantly on the immature end of the differentiation trajectory – conceivably provides a larger available pool of BCAS1+ cells at early recovery, tipping the scales in favor of differentiation. Given that deep cortical ASPA+ oligodendrocytes recovered to baseline levels at late recovery only in PLX‐treated mice, but not cuprizone‐only treated mice (Figure 5), and late recovery oligodendrocytes are predominantly represented by newly formed cells (Orthmann‐Murphy et al. 2020), we suspect that many of these early recovery BCAS1+ cells formed in PLX‐treated mice become stably incorporated into the late recovery cortex. This finding suggests that deep cortical reactive microglia impair deep cortical oligodendrocyte regeneration during recovery from demyelination by blocking OPCs from transitioning to a BCAS1+ state.

Notably, this interpretation does not preclude the possibility that pre‐existing oligodendrocytes survive or are dying with delayed clearance when cortical reactive microglia are depleted. In fact, there are likely overlapping pathogenic roles of cortical reactive microglia, especially at early recovery (Orthmann‐Murphy et al. 2020). The increased number of ASPA+ cells at early recovery could represent dying, surviving, and newly formed oligodendrocytes. However, neither pre‐existing nor dying oligodendrocytes would be expected to express BCAS1+. In future studies, fate‐tracing approaches of oligodendrocyte lineage cells in PLX‐treated mice should be able to parse out the relative contributions of surviving baseline oligodendrocytes and newly formed oligodendrocytes to the final tally of ASPA+ oligodendrocytes in deep cortex at late recovery. These approaches will also determine whether there is delayed clearance of pre‐existing or BCAS1+ differentiating oligodendrocytes when reactive microglia are depleted during recovery.

Our finding that cortical reactive microglia impair oligodendrocyte differentiation post‐cuprizone is unexpected, as previous studies of white matter demyelination models showed that reactive microglia can support oligodendrocyte regeneration (Baaklini et al. 2023; Miron et al. 2013). For example, using the lysolecithin‐induced model of demyelination, Miron et al. (2013) identified a subset of microglia present during the remyelination stage that expressed ARG1 + IGF1 + CD206+; these microglia likely secrete factors that promote OPC differentiation as their conditioned media promoted OPC differentiation in vitro, and their selective depletion in vivo impaired remyelination. Similarly, Baaklini et al. (2023) showed that microglia depletion after lysophosphatidylcholine‐induced spinal cord demyelination led to reduced OPC recruitment, proliferation, and differentiation. Together, these studies indicate that white matter microglia can adopt pro‐regenerative reactive states. Although we found that depleting cortical microglia led to increased numbers of deep cortical BCAS1+ and ASPA+ cells at early recovery, a few deep cortical reactive microglia survived in PLX‐treated mice (Figure 3B,C). An alternative possibility to explain our findings is that the surviving deep cortical microglia in PLX‐treated mice are pro‐regenerative. It is also possible that individual Csf1r inhibitors have unexplored off‐target effects on the oligodendrocyte lineage post‐cuprizone. Future studies are needed to further characterize the role of surviving reactive microglia under Csf1r inhibitor treatment conditions, and identify potential unexpected effects of these compounds on oligodendrocyte lineage cells directly.

How could deep cortical reactive microglia reduce the number of newly forming oligodendrocytes? Their amoeboid morphology and upregulation of CD68 are reminiscent of the previously characterized early developmental subset of microglia that phagocytose OPCs prior to the onset of myelination in the corpus callosum (Irfan et al. 2022). In fact, microglial phagocytosis of OPCs and oligodendrocytes during early postnatal development in white matter limits the number of oligodendrocytes and myelin sheaths formed (Nemes‐Baran et al. 2020). It is possible, then, that deep cortical reactive microglia are phagocytosing pre‐myelinating BCAS1+ oligodendrocytes. While this function is developmentally necessary, it would be detrimental to oligodendrocyte recovery after demyelination. Another possibility is that deep (but not superficial) cortical reactive microglia release pro‐inflammatory cytokines that could be directly toxic to newly forming oligodendrocytes and/or induce the recruitment of other cytotoxic immune cells (Lee et al. 2018; Wang et al. 2018; Wies Mancini et al. 2023) or induce other local cells, such as astrocytes, to become directly toxic to oligodendrocytes (Guttenplan et al. 2020; Liddelow et al. 2017).

It is unlikely that the depletion of microglia alone will be a sufficient therapeutic intervention to promote oligodendrocyte recovery following inflammatory demyelinating attacks in MS, given the distinct roles of microglia in white and gray matter lesions. Our findings indicate that the depletion of cortical reactive microglia post‐cuprizone increased the available pool of early differentiating BCAS1+ cells. We previously showed in the healthy adult cortex that most differentiating oligodendrocytes do not survive (Hughes et al. 2018). Future studies will determine whether additional interventions are required to promote the final stages of oligodendrocyte differentiation and successful integration of mature cortical oligodendrocyte post‐demyelination. Ultimately, targeting specific reactive microglia functions at key time‐points to promote oligodendrocyte regeneration could prevent axonal damage and progressive disease in MS.

## Author Contributions

H.K.L. and J.L.O.‐M. conceptualized and designed the study. H.K.L., J.G., G.E.P., C.M., and T.M. carried out the methodology, investigations, and data analyses. H.K.L. and J.L.O.‐M. prepared the figures and wrote and edited the manuscript. All authors reviewed and approved the final manuscript.

## Funding

This work was funded by the National Institute of Neurological Disorders and Strokes Award Number R01NS132793 to JOM; National Multiple Sclerosis Society Award Number RG‐2207‐40022 to JOM; the Jill and Mark Fishman Foundation to JOM, the Linda Pechenik Montague Investigator Award to JOM, and the National Science Foundation Graduate Research Fellowship Program DGE‐1845298 to HKL.

## Ethics Statement

This study was approved by the Institutional Animal Care and Use Committee at the University of Pennsylvania, Protocol # 806627.

## Conflicts of Interest

J.L.O‐.M. is a consultant for Vigil Neuroscience, Savanna Bio, and Ionis Pharmaceuticals. J.L.O‐.M. was site PI for clinical trials for Vigil Neuroscience.

## Supporting information


**Appendix S1:** Supplementary information.

## Data Availability

All data generated or analyzed in this study are included in the manuscript. Data will be made available upon request to authors. On publication, study data will be deposited in Dryad (imaging data) and Gitlab (analysis code).
