# Supplementary material for: Depletion of Microglia Increases Cortical Oligodendrocyte Density During Remyelination
Source: Glia. 2026 Jan 8;74(3):e70120. doi: 10.1002/glia.70120 (PMC12784134; doi:10.1002/glia.70120)
Supplement: Supplementary file 1 — Appendix S1: Supplementary information. [file GLIA-74-0-s001.pdf]

Supplementary Table: Key Reagents

Reagent type (species) or resource	Designation	Source or reference	Identifiers	Additional Information
Stain, strain background (*M. musculus)*	STOCK Tg(Mobp-EGFP) IN1Gsat/Mmucd. "Mobp-eGFP"	MMRC	RRID: MGI:5003916	(Orthmann-Murphy et al., 2020)
Stain, strain background (*M. musculus)*	B6.129P2(Cg)-*Cx3cr1*^{tm1Litt}/J. "Cx3Cr1-eGFP"	The Jackson Laboratory	RRID:IMSR_JAX:005582	
Stain, strain background (*M. musculus)*	C57BL/6	Charles River	RRID:IMSR_CRL:27	
Antibody	ASPA	GeneTex	RRID:AB_2036283	1:1500
Antibody	CC3	Cell Signaling Technology	RRID:AB_2070042	1:200
Antibody	GFAP	Agilent (DAKO)	RRID:AB_10013382	1:500
Antibody	GFP	Aves	RRID:AB_10000240	1:4000
Antibody	Iba1	Wako	RRID:AB_839504	1:300
Antibody	CD68	Biolegend	RRID:AB_2044003	1:500
Antibody	P2RY12	Anaspec	RRID:AB_2298886	1:500
Antibody	NG2	Millipore	RRID:AB_91789	1:200
Antibody	BCAS1 (Rb)	Synaptic Systems		1:1000
Antibody	MBP	Aves Labs	RRID:AB_2313550	1:500
Antibody	Donkey Anti-Rabbit IgG conjugated to Alexa 488, Alexa 594, or Alexa 647	Jackson Immuno	RRID:AB_2340619<br>RRID:AB_2340621<br>RRID:AB_2492288	1:500
Antibody	Donkey Anti-Mouse IgG conjugated to Alexa 488, Alexa 594, or Alexa 647	Jackson Immuno	RRID:AB_2341099<br>RRID:AB_2340855<br>RRID:AB_2340863	1:500

Antibody	Donkey Anti-Chicken IgG conjugated to Alexa 488, Alexa 594, or Alexa 647	Jackson Immuno	RRID:AB_2340375<br>RRID:AB_2340377<br>RRID:AB_2340379	1:500
Antibody	Donkey Anti-Rat IgG conjugated to Alexa 647	Jackson Immuno	RRID:AB_2338393	1:500
Probe	Mm-Tmem119 (C3)	ACD Bio	Catalog #472901-C3	
Probe	Mm-Tmem119 (C1)	ACD Bio	Catalog #472901	
Dye	Opal 570	ACD Bio	Catalog #SKU FP1488001KT	1:1500
Dye	Opal 690	ACD Bio	Catalog #SKU FP1497001KT	1:1500
Chemical compound, drug	bis(cyclohexanone) oxaldihydrazone (Cuprizone)	Santa Cruz Biotechnology	sc-217769	
Chemical compound, drug	5kg AIN-76A Diet With 290 mg PLX3397, irradiated	SelleckChem (PLX3397), Research Diets (AIN-76A Diet)	SelleckChem: S7818; Research Diets: D15112401i	
Chemical compound, drug	1.5kg AIN-76A Rodent diet with 1200ppm PLX5622, irradiated	SelleckChem (PLX5622), Research Diets (AIN-76A Diet)	SelleckChem: S8874; Research Diets: D23051705i	
Software/algorithm	Fiji	http://fiji.sc	RRID:SCR_002285	
Software/algorithm	ImageJ	https://imagej.nih.gov/ij/	RRID:SCR_003070	
Software/algorithm	Prism	https://www.graphpad.com/features	RRID:SCR_002798	

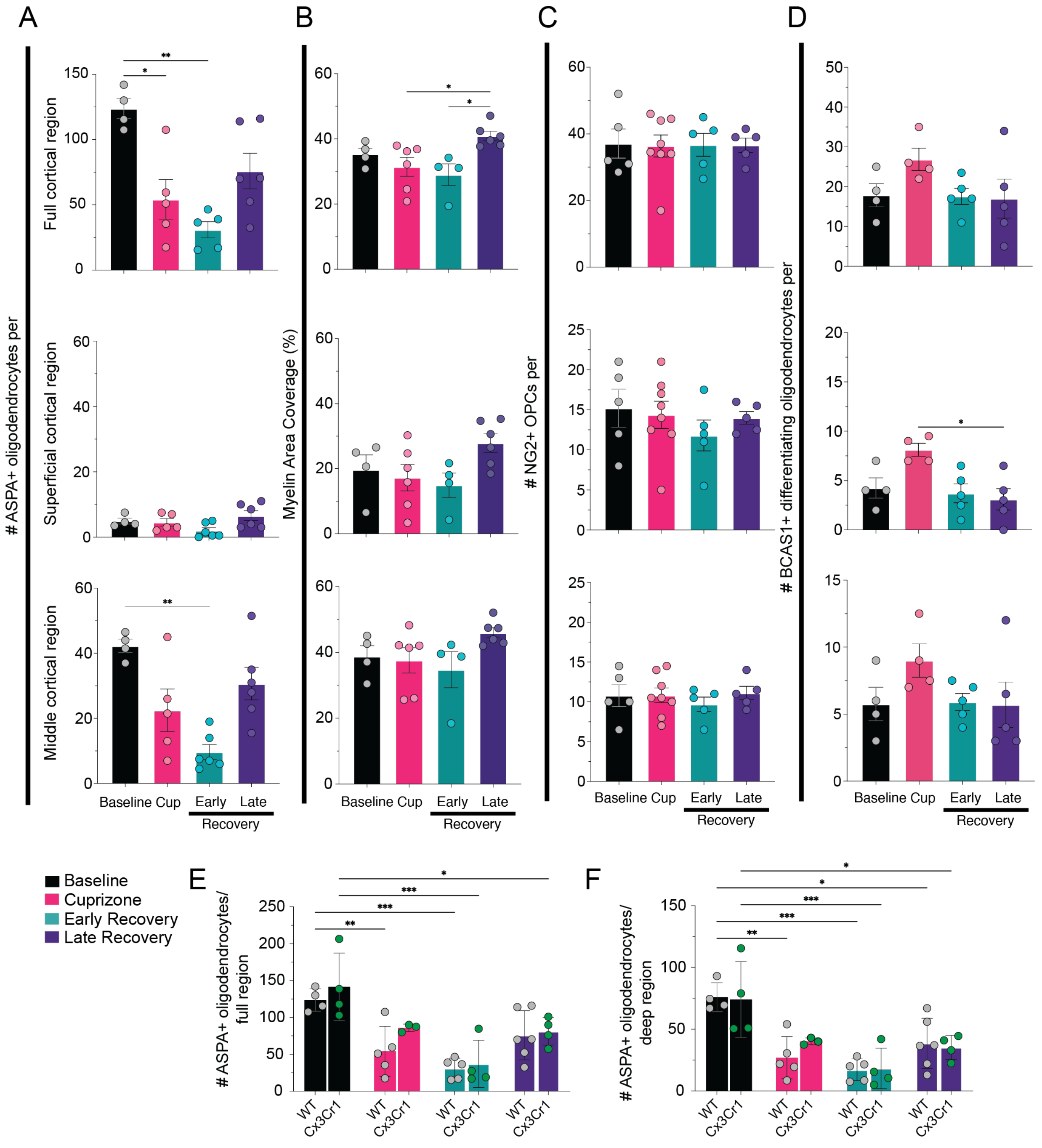

Supplementary Figure 1. Replacement of oligodendrocytes in superficial and middle cortical regions is not impaired. Quantification of the total number of ASPA+ oligodendrocytes (A), MBP+ Myelin Area Coverage percentage (B), and the number of NG2+ OPCs (C), and BCAS1+ differentiating oligodendrocytes (D) in the full somatosensory cortical region (0 μm – 750 μm, top row), the superficial cortical region (0 μm – 250 μm, middle row) and middle cortical region (250 μm – 500 μm, bottom row) at baseline, after 4 weeks of cuprizone-treatment (Cup), and at early and late recovery. E-F: Quantification

of the total number of ASPA+ oligodendrocytes in WT (either Mobp-eGFP or WT littermate controls) and Cx3Cr1-eGFP mice at each time-point in the full (E) and deep (F) cortical region. Significant comparisons are A: Full Region: **Baseline** mice (n=4) vs. **Cuprizone** mice (n=5), p = 0.0201; **vs. Early Recovery** mice (n=5), p = 0.0012. Middle Region: **Baseline** mice (n=4) **vs. Early Recovery** mice (n=5)**,** p=0.0056. B: Full Region: **Late Recovery** mice (n=6) **vs. Early Recovery** mice (n=4), p=0.0187; **vs. Cuprizone** mice (n=6), p=0.0384. D: Superficial Region: **Cuprizone** mice (n=4) **vs. Late Recovery** mice (n=5), p=0.0353. E: **WT Baseline** (n=4) **vs. WT Cuprizone** (n=5), p=0.0061; **vs. WT Early Recovery** (n=5), p=0.0003; **Cx3Cr1 Baseline** (n=4) **vs. Cx3Cr1 Early Recovery** (n=4), p=0.0001; **vs. Cx3Cr1 Late Recovery** (n=4), p=0.0284. F: **WT Baseline** (n=4) **vs. WT Cuprizone** (n=3), p=0.0011; **vs. WT Early Recovery** (n=4), p=0.0001; **vs. WT Late Recovery** (n=6), p=0.0108; **Cx3Cr1 Baseline** (n=4) **vs. Cx3Cr1 Early Recovery** (n=4), p=0.0005; **vs. Late Recovery** (n=4), p=0.0170. Significant comparisons for WT ASPA quantification are shown in Panel A and Figure 1C. Statistical comparisons are one-way ANOVA with Tukey correction for A-D, two-way ANOVA with Tukey correction for E,F. Horizontal bar represents mean value; error bars, standard error of the mean. Dots indicate the number of mice per condition; in panels E-F, green dots are Cx3Cr1-eGFP mice and gray dots are WT mice (Mobp-eGFP or WT littermate controls).

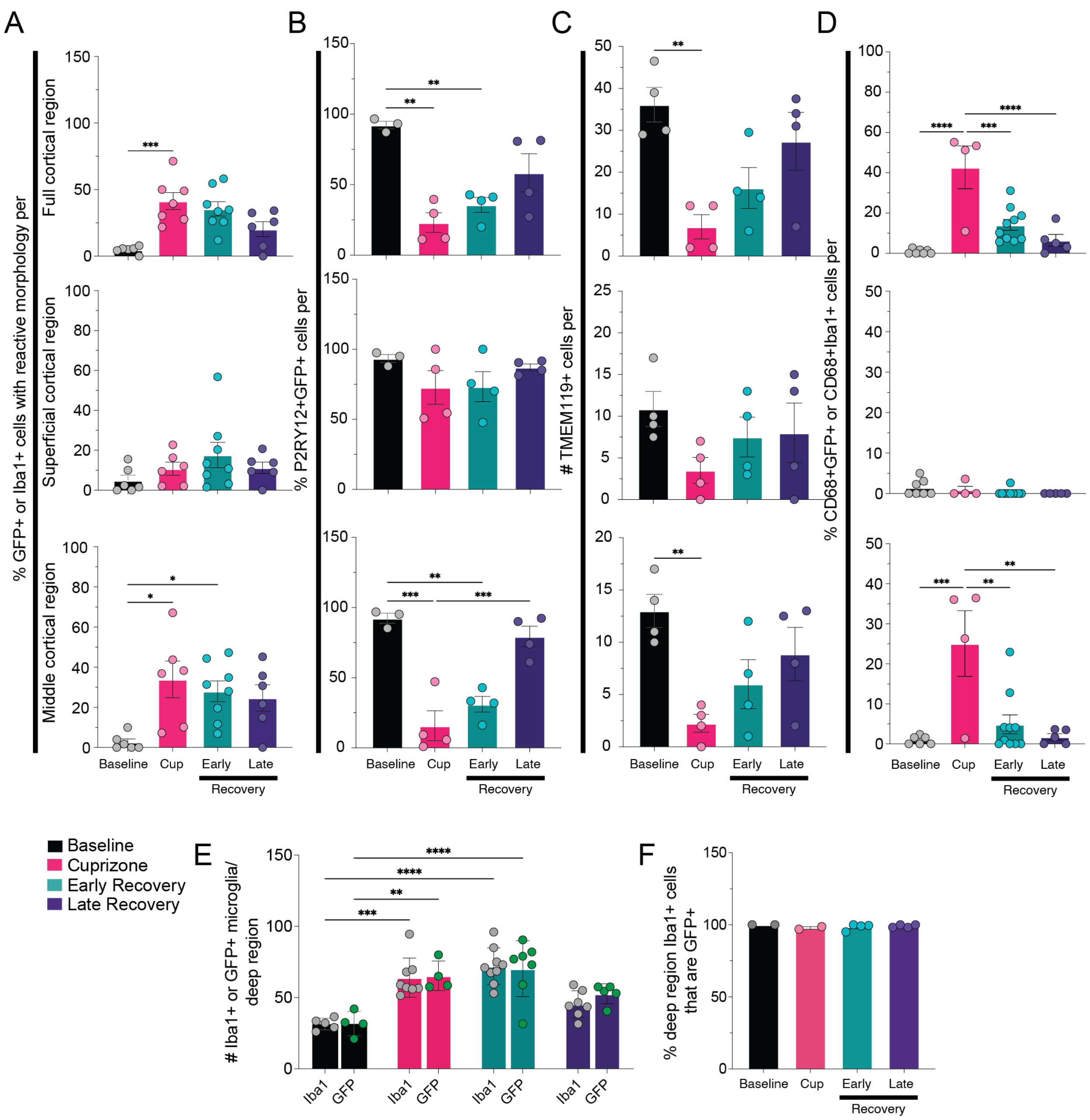

Supplementary Figure 2. Cuprizone-induced demyelination differentially alters cortical microglia reactive state in superficial and deep cortical regions. A-D: Quantification of the proportion of reactive morphology Iba1+ or GFP+ microglia (A), the proportion of GFP+ cells that are P2RY12+ (B), the number of Tmem119+ cells (C) and the proportion of Iba1+ or GFP+ cells that are CD68+ (D) in the full somatosensory cortical region (0 µm – 750 µm, top row), the superficial region (0 µm – 250 µm, middle row), and middle region (250 – 500 µm, bottom row) at baseline, after cuprizone treatment (Cup) and after two (Early) or five (Late) weeks of recovery. Significant comparisons are A: Full Region: **Baseline** mice (n=6) **vs. Cuprizone** mice (n=7), p=0.0004; Middle Region: **Baseline** mice (n=6) **vs. Cuprizone**

mice (n=7), p=0.0001; **vs. Early Recovery** mice (n=8), p=0.0004. B: Full Region: **Baseline** mice (n=3) **vs. Cuprizone** mice (n=4), p=0.0011; **vs. Early Recovery** mice (n=4), p=0.0052; **vs. Late Recovery** mice (n=4), p=0.0952. Middle Region: **Baseline** mice (n=3) **vs. Cuprizone** mice (n=4), p=0.0002; **vs. Early Recovery** mice (n=4), p=0.0011; **Cuprizone** mice (n=4) **vs. Late Recovery** mice (n=4), p=0.0004. C: Full Region: **Baseline** mice (n=4) **vs. Cuprizone** mice (n=4), p=0.006; **vs. Early Recovery** mice (n=4), p=0.0611; Middle Region: **Baseline** mice (n=4) **vs. Cuprizone** mice (n=4), p=0.0099. D: Full Region: **Cuprizone** mice (n=4) **vs. Baseline** mice (n=7), p<0.0001; **vs. Early Recovery** mice (n=10), p=0.0004; **vs. Late Recovery** mice (n=5), p<0.0001; Middle Region: **Cuprizone** mice (n=4) **vs. Baseline** mice (n=7), 0.0007; **vs. Early Recovery** mice (n=10), 0.0018; **vs. Late Recovery** mice (n=5), p=0.0014. E: Comparison of the number of Iba1+ cells in WT mice (Mobp-eGFP mice or WT littermates, gray dots) to GFP+ cells in Cx3Cr1-eGFP mice (green dots) at each time-point. There is no significant difference between the number of Iba1+ and GFP+ cells at any time-point. Significant comparisons are **Iba1 Baseline** mice (n=5) **vs. Iba1 Cuprizone** mice (n=8), p=0.0002; **vs. Early Recovery** mice (n=9), p<0.0001; **GFP Baseline** mice (n=4) **vs. GFP Cuprizone** mice (n=4); p=0.0023; **vs. GFP Early Recovery** mice (n=4), p<0.0001. F. Quantification of the proportion of deep cortical Iba1+ cells that are also GFP+ in Cx3Cr1-eGFP mice. Statistical comparisons are one-way ANOVA with Tukey correction for A-D, F, and two-way ANOVA with Tukey correction for E. Horizontal bar represents mean value; error bars, standard error of the mean. Dots indicate the number of mice per condition; in panel E, green dots are Cx3Cr1-eGFP mice and gray dots are WT mice (Mobp-eGFP or WT littermate controls).

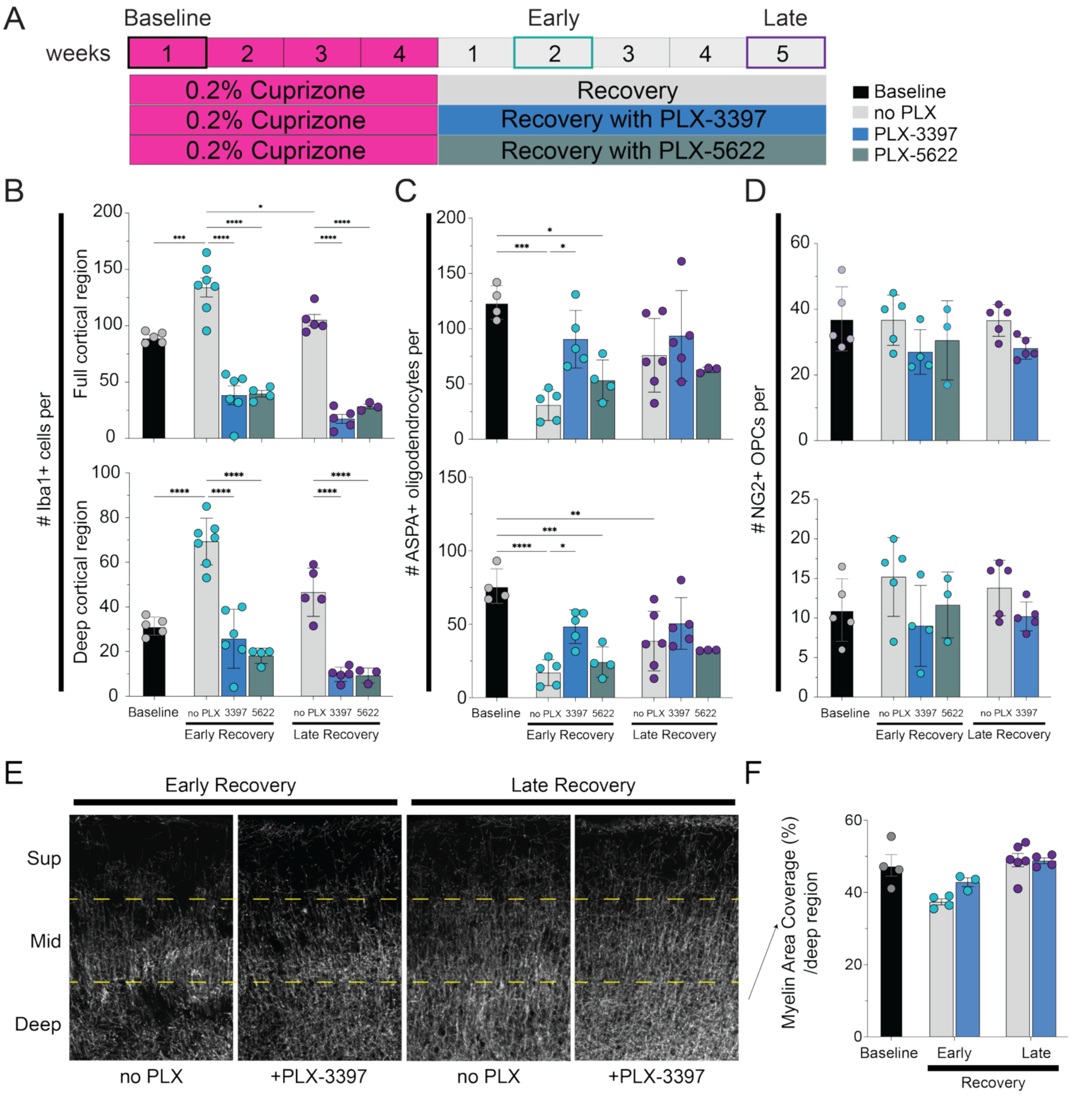

Supplementary Figure 3. Comparison of the effect of two different Csf1r inhibitors on cortical microglia depletion and oligodendrocyte density. A. Experimental paradigm with administration of Csf1r antagonists PLX-3977 (3397, blue) or PLX-5622 (5622, sage) post-cuprizone administration to deplete reactive microglia during recovery. B-D: Quantification of the number of Iba1+ microglia (B), ASPA+ oligodendrocytes (C), and NG2+ OPCs (D) in the full somatosensory cortical region relative to the pial surface (0 µm - 750 µm, top row), and the deep cortical region (500 µm - 750 µm, bottom row) in mice treated with cuprizone followed by recovery alone (No PLX, grey), or recovery with treatment with PLX-3397 (blue) or PLX-5622 (sage). Significant comparisons are B: Full Region: **Baseline** mice (n=5) **vs. Early Recovery no PLX** mice (n=7), p= 0.0004; **Early Recovery no PLX** mice (n=7) **vs. Early Recovery + PLX-3397** mice (n=6), p<0.0001; **vs. Early Recovery + PLX-5622** mice (n=4), p<0.0001; **vs. Late Recovery no PLX** mice (n=5), p=0.0364; **Late Recovery no PLX** mice (n=5) **vs. Late Recovery + PLX-**

3397 mice (n=5), p<0.0001; **vs. Late Recovery + PLX-5622** mice (n=3), p<0.0001; Deep Region: **Baseline** mice (n=5) **vs. Early Recovery no PLX** mice (n=7), p<0.0001; **Early Recovery no PLX** mice (n=7) **vs. Early Recovery + PLX-3397** mice (n=6), p<0.0001; **vs. Early Recovery + PLX-5622** mice (n=4), p<0.0001; **Late Recovery no PLX** mice (n=5) **vs. Late Recovery + PLX-3397** mice (n=5), p<0.0001; **vs. Late Recovery + PLX-5622** mice (n=3), p<0.0001. C: Full Region: **Baseline** mice (n=4) **vs. Early Recovery no PLX** mice (n=5), p=0.0004; **vs. Early Recovery + PLX-5622** mice (n=4), p=0.0137; **Early Recovery no PLX** mice (n=5) **vs. Early Recovery + PLX-3397** mice (n=5), p=0.0222; Deep Region: **Baseline** mice (n=4) **vs. Early Recovery no PLX** mice (n=5), p<0.0001; **vs. Early Recovery + PLX-5622** mice (n=4), p=0.0003; **vs. Late Recovery no PLX** mice (n=6), p=0.0052; **Early Recovery no PLX** mice (n=5) **vs. Early Recovery + PLX-3397** mice (n=5), p=0.023. E- Representative images of MBP+ myelin following two (early) and five (late) weeks of recovery with and without PLX-3397 administration. F. Quantification of the myelin area coverage based on MBP signal in healthy adult mice after two and five weeks following 4 weeks of cuprizone administration (gray) or with PLX-3397 treatment (blue) in the deep region. Statistical comparisons are one-way ANOVA with Tukey correction. Horizontal bar represents mean value; error bars, standard error of the mean. Dots indicate the number of mice per condition, teal dots represent early recovery and purple dots represent late recovery.

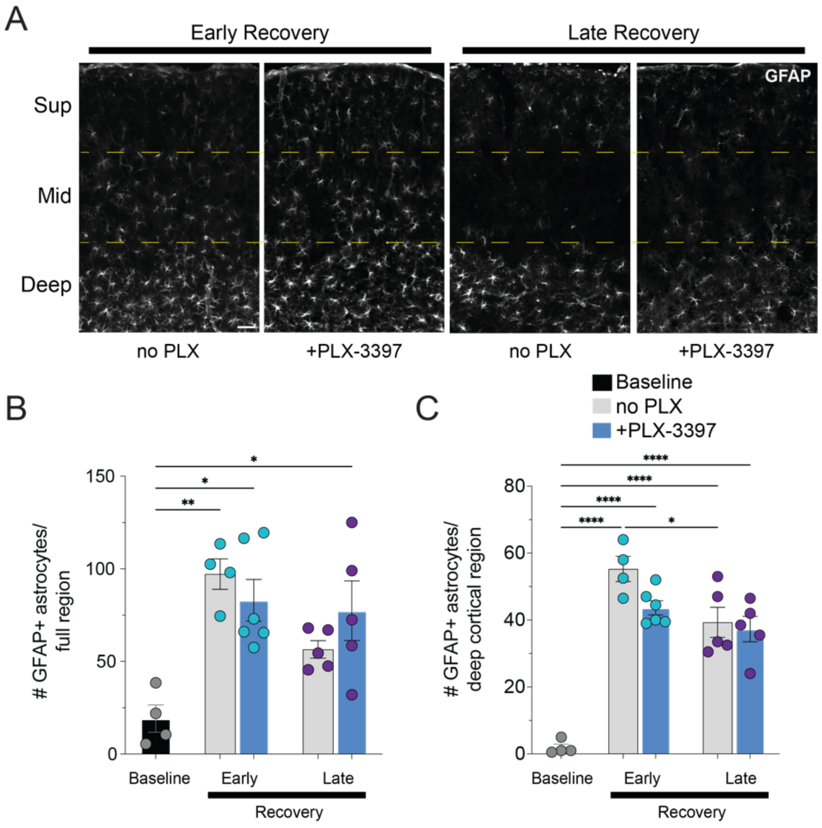

Supplementary Figure 4. Deep cortical GFAP+ cortical reactive astrocytes are persistently elevated during recovery, with or without Csf1r inhibitor treatment. A. Representative images of GFAP+ reactive astrocytes at early and late recovery, without (No PLX) or in the presence of PLX-3397 (+PLX-3397). Dashed yellow lines denote superficial (Sup, 0 µm – 250 µm), middle (Mid, 250 µm – 500 µm) and deep (500 µm – 750 µm) cortical regions relative to the pial surface. Scale bar, 50 µm. B. Quantification of the number of GFAP+ reactive astrocytes in the full somatosensory cortical region relative to the pial surface (0 µm - 750 µm, left), and deep cortical region (500 µm - 750 µm, right) in mice treated with cuprizone followed by recovery (No PLX, grey) or treatment with PLX-3397 (blue). B: Significant comparisons are B: Full region: **Baseline** mice (n=4) **vs. Early Recovery no PLX** mice (n=4), p=0.0049; **vs. Early Recovery + PLX-3397** mice (n=6), p=0.0132; **vs. Late Recovery + PLX-3397** mice (n=5), p=0.0362. C: Deep region: **Baseline** mice (n=4) **vs. Early Recovery no PLX** mice (n=4), p<0.0001; **vs. Early Recovery + PLX-3397** mice (n=6), p<0.0001; **vs. Late Recovery no PLX** mice (n=5), p<0.0001; **vs. Late Recovery + PLX-3397** mice (n=5), p<0.0001; **Early Recovery no PLX** mice (n=4) **vs. Late Recovery no PLX** mice (n=5), p=0.0351. Statistical comparisons are one-way ANOVA with Tukey correction. Horizontal bar represents mean value; error bars, standard error of the mean. Dots indicate the number of mice per condition.
